# Small steps forward: Adjunctive therapy for T1D

**DOI:** 10.1111/1753-0407.13326

**Published:** 2022-10-07

**Authors:** Zachary Bloomgarden, Desmond Schatz

**Affiliations:** ^1^ Department of Medicine, Division of Endocrinology, Diabetes and Bone Disease Icahn School of Medicine at Mount Sinai New York New York USA; ^2^ Diabetes Institute, University of Florida College of Medicine Gainesville Florida USA

An important observation is that features of type 2 diabetes (T2D) are now being seen in many individuals with type 1 diabetes (T1D). In a 6‐year follow‐up of the Diabetes Complications and Control Trial (DCCT), among those initially randomized to intensive glycemic management, approximately 10% had a positive family history of T2D, with final triglyceride level 92 versus 76, low‐density lipoprotein cholesterol 118 versus 111, and apolipoprotein B 90 versus 82 mg/dl among those not having such a history, and these individuals gained more weight during the trial, with final body mass index (BMI) 27.8 versus 26.4 kg/m^2^, respectively.^1^ Hypoglycemia is usually treated by ingestion of rapidly absorbed carbohydrate, and as a consequence frequent low glucose may contribute to weight gain; in the DCCT, those in the lowest tertile of hypoglycemia frequency had less weight gain than those in the second and third tertiles.^2^ Higher glycosylated hemoglobin (HbA1c) during the DCCT was also associated with greater weight gain.^2^ Weight gain appears to be a mediator of adverse outcome of glycemic treatment, and in a different analysis of DCCT participants randomized to intensive glycemic management, of those in the highest quartile of weight gain, 22% and 48% met the metabolic syndrome criterion for excess waist circumference at DCCT closeout and after 6 years of follow‐up, respectively, while just 3% and 14% of those in the first through third quartiles of weight gain had excess waist circumference at these times. Furthermore, those in the highest weight gain quartile had higher HbA1c, insulin dose, and levels of triglyceride, non–high‐density lipoprotein cholesterol, and blood pressure, with this degree of weight gain showing significant association with carotid intima‐medial thickness, a marker of atherosclerotic cardiovascular disease (ASCVD).^3^

Based on these observations, it has been a concern that individuals with T1D are increasingly likely to be overweight or obese. This was observed in follow‐up of the Pittsburgh Epidemiology of Diabetes Complications (EDC) Study prospective cohort from 1986 to 2007.^4^ The T1D Exchange Clinic Registry reports that among their population of 22 697 persons with T1D treated from 2016 to 2018, 42% of adolescents, 48% of those ages 18–25, and 69% of those age 26 and older were overweight or obese.^5^ A number of complications are associated with insulin resistance. Approximately one quarter of the US population has evidence of nonalcoholic fatty liver disease (NAFLD), seen in association with greater body weight,^6^ and it should not therefore be surprising that NAFLD is now being reported with increasing frequency in T1D. A meta‐analysis of 20 studies of 3901 persons with T1D reported an overall NAFLD prevalence of 19% on imaging studies,^7^ with recent imaging studies using hepatic elastography reporting NAFLD prevalence of 20%^8^ and 47%^9^ among persons with T1D. A study of 4899 persons with T1D with indices based on age, BMI, and laboratory test results suggested NAFLD prevalence as high as 71%.^10^

Analysis of ASCVD events among 36 869 persons with T1D at median age 35 matched 1:5 with controls shows 6‐fold greater likelihood of CVD mortality and 5‐fold greater likelihood of CVD hospitalization.^11^ The mechanism of the association of greater body weight with adverse consequence in T1D is likely to be insulin resistance, with insulin‐resistant persons with T1D showing marked increase in development of coronary disease in comparison to those who are insulin sensitive.^12^, ^13^, ^14^ Using the waist circumference, HbA1c, and blood pressure to derive an estimate of the glucose disposition rate (eGDR), several groups have shown lower eGDR to be associated with ASCVD and mortality,^15^, ^16^ and using such an approach to estimate the change in insulin sensitivity over time in 1375 participants in the DCCT at entry and at 18.5‐year follow‐up, those with lower insulin sensitivity at both time points had greater likelihood of development of CVD events.^17^

Although insulin therapy is the mainstay of treatment of T1D to attain glucose control, and although glycemia is certainly the most important predictor of outcome among persons with T1D, intensive therapy may, then, compound weight gain, in turn leading to a variety of conditions associated with insulin resistance and adverse outcome. Consideration of measures to reduce weight gain in T1D is therefore important including counseling on healthy (and weight reducing) nutritional programs and adequate physical activity. Specifying meal times rather than allowing “grazing and snacking” may be a simple measure to improve glycemic control.^18^ Ketogenic diets restricting dietary carbohydrate to as little as 20–70 g/day have been suggested for weight control, and may be associated with reduction in HbA1c and daily insulin dose in adults, but this may occur at the expense of insufficient insulin, fatigue, dyslipidemia, and/or increased hypoglycemic events, and such approaches are not appropriate for youth with T1D who require carbohydrates for growth.^19^, ^20^ In a T1D population, 11 357 inactive persons were compared with 3459 and 3212 participating in physical activity 1–2 times and >2 times weekly; the former had higher BMI, HbA1c, and triglyceride levels and required higher daily insulin dosage than the latter two groups.^21^

A number of T2D treatment approaches have been considered for potential use in patients with T1D. A meta‐analysis of five studies of a total of 185 persons with T1D randomized to adjunctive metformin showed significant reduction in daily insulin dosage but not in HbA1c level,^22^ although a more recent review of five clinical trials of metformin failed to show convincing evidence of either effect.^23^ None of these studies, however, focused on obese persons with T1D, and one such study, although not showing reduction in HbA1c, did show a greater degree of weight loss and lesser likelihood of weight gain among participants randomized to metformin.^24^ Metformin also improved insulin sensitivity in a controlled trial of 37 obese or overweight youth with T1D,^25^ and a recent controlled trial of 50 adolescents with T1D and baseline A1c averaging ~10% showed significantly greater HbA1c reduction with metformin.^26^ The insulin sensitizer rosiglitazone was studied in T1D and was found to lead to significant HbA1c reduction in obese T1D, although not in those with BMI < 30 kg/m^2^.^27^

A study of 296 persons with T1D showed that pramlintide was associated with reduction in postprandial glucose excursions and body weight, although with similar HbA1c reduction to that seen in placebo‐treated persons.^28^ Studies of the bile acid sequestrant colesevelam^29^ and of the dipeptidyl peptidase IV inhibitor sitagliptin^30^, ^31^ also failed to show improvement in glycemia. The glucagon‐like peptide 1 receptor agonist (GLP‐1RA) liraglutide was, however, associated with weight loss in a randomized trial of 100 persons with T1D,^32^ and a recent observational study of 54 persons with T1D receiving once‐weekly GLP‐1RA showed significant reduction in HbA1c, weight, and continuous glucose‐monitoring parameters.^33^

There has been considerable interest in the use of sodium glucose cotransporter 2 inhibitors (SGLT2i) in T1D treatment. The potential benefits of SGLT2i in reduction in diabetic kidney disease, in NAFLD, and in heart failure reported in T2D have provoked particular interest in their use in T1D. Randomized controlled trials of treatment with dapagliflozin, empagliflozin, and sotagliflozin including >6000 participants with T1D have shown reductions in HbA1c, fasting and postprandial glucose, body weight, and blood pressure, although with side effects including genital mycotic infections.^34^, ^35^, ^36^, ^37^ A particularly worrisome complication associated with use of SGLT2i, diabetic ketoacidosis (DKA), may occur at normal or only mildly elevated glucose levels, so‐called euglycemic ketoacidosis.^38^ A meta‐analysis of 18 placebo‐controlled trials of SGLT2i in T1D including 7396 participants over a mean duration of 19 weeks showed a 2.81‐fold increased risk of DKA, affecting 1%–4% of treated patients, with greatest risk of DKA in trials of persons with lower insulin sensitivity and higher body weight, particularly in association with excessive insulin dose reduction,^39^ so that caution in use of these agents is appropriate.

How, then, should we interpret these studies? Prevalence rates of overweight and obesity are increasing in T1D, leading to insulin resistance, and at least one tenth of persons with T1D have characteristics of T2D. This observation leads to increasing interest in the use of T2D treatment approaches in the management of persons with T1D. The development of criteria to determine which persons with T1D are appropriate candidates for which T2D treatments will be important. While we await further studies in this area, shared decision‐making in which clinicians and patients work together to understand and carefully use such approaches may be appropriate for selected patients.
